# The Deformation of Polydimethylsiloxane (PDMS) Microfluidic Channels Filled with Embedded Circular Obstacles under Certain Circumstances

**DOI:** 10.3390/molecules21060798

**Published:** 2016-06-18

**Authors:** Changhyun Roh, Jaewoong Lee, Chankyu Kang

**Affiliations:** 1 Biotechnology Research Division, Advanced Radiation Technology Institute (ARTI), Korea Atomic Energy Research Institute (KAERI), 29 Guemgu-gil, Jeongeup, Jeonbuk 56212, South Korea; chroh@kaeri.re.kr; 2 Department of Textile Engineering & Technology, Yeungnam University, 280 Daehak-Ro, Gyeongsan, Gyeongbuk 38541, South Korea; jaewlee@yu.ac.kr; 3 Ministry of Employment and Labor, Center for Major Industrial Accident Prevention, 34 Yeosusandan-ro, Yeosu-si, Jeollanam-do 59361, South Korea

**Keywords:** PDMS bulging, ANSYS Workbench, embedded obstacles, steady-state flow, pressure drop, flow velocity

## Abstract

Experimental investigations were conducted to determine the influence of polydimethylsiloxane (PDMS) microfluidic channels containing aligned circular obstacles (with diameters of 172 µm and 132 µm) on the flow velocity and pressure drop under steady-state flow conditions. A significant PDMS bulging was observed when the fluid flow initially contacted the obstacles, but this phenomenon decreased in the 1 mm length of the microfluidic channels when the flow reached a steady-state. This implies that a microfluidic device operating with steady-state flows does not provide fully reliable information, even though less PDMS bulging is observed compared to quasi steady-state flow. Numerical analysis of PDMS bulging using ANSYS Workbench showed a relatively good agreement with the measured data. To verify the influence of PDMS bulging on the pressure drop and flow velocity, theoretical analyses were performed and the results were compared with the experimental results. The measured flow velocity and pressure drop data relatively matched well with the classical prediction under certain circumstances. However, discrepancies were generated and became worse as the microfluidic devices were operated under the following conditions: (1) restricted geometry of the microfluidic channels (*i.e.*, shallow channel height, large diameter of obstacles and a short microchannel length); (2) operation in quasi-steady state flow; (3) increasing flow rates; and (4) decreasing amount of curing agent in the PDMS mixture. Therefore, in order to obtain reliable data a microfluidic device must be operated under appropriate conditions.

## 1. Introduction

Microfluidic devices have attracted considerable attention for many evolutional aspects of applied sciences and engineering because of their actuated controls of micro-scale fluid behaviors in miniaturized devices [1,2]. In the advance of micro-electronics fabrication and biotechnologies, microfluidic devices have become the preferable choices in several fields of application [3]. On the other hand, an understanding of liquid flow through microchannels or microstructures plays a key role in the accurate and economic operation of microfluidic devices [4]. Microfluidic devices require highly accurate low- liquid flowmeters operating in the microliter and nanoliter per minute range for use in various microfluidic applications. Microfluidic channels integrated with embedded obstacles offer the following advantages compared to plain microfluidic channels: (i) enhanced heat and mass transfer coefficients [5,6,7,8]; (ii) increased mixing efficiency [9,10,11]; and (iii) they allow chemotaxis gradients [12,13]. Therefore, fluid flow in porous media on the micro-scale has been applied in micro-reactors [14], micro-separators [15], micro-heat exchangers [16], micro-pumps [17], and micro-filters [18]. Despite their widespread applications, increasing the shear force near microstructures in porous media leads to augmented pressure drops [19]. Therefore, the optimal design of the microstructure-integrated microchannel assembly in microfluidic systems will increase its efficiency.

Many studies examining fluid flow in microfluidic channels have operated under the following conditions: fully developed, steady-state and incompressible flows. Among them, several studies using rigid microfluidic channels reported significantly higher values of the friction constant (*f·Re*) for microfluidic channels than those observed for classical channels [20,21].The decreased channel aspect ratio (α) and the channel height (h) to width (w) ratio in rectangular microfluidic channels increased the friction factor significantly [22]. Some studies have suggested that the unexpectedly large discrepancies from the predicted values with respect to the friction factor (*f*) data were due to experimental uncertainties, microchannel geometry and surface roughness [23]. Recently, a transient filling fluid through long, shallow microfluidic channels containing periodically spaced circular obstacles with two different diameters (*i.e.*, 132 µm and 172 µm) showed that quasi-steady flow was observed with undesired PDMS bulging due to the applied flow rates that generated large deviations from the predicted values [24]. Although the controversy regarding the validity of the laws of hydrodynamics in microchannels can now be considered an outdated issue, the reliability of PDMS-based microfluidic devices is still a significant topic due to the popularity of PDMS-based microfluidic systems [25,26,27,28,29].

Polydimethylsiloxane (PDMS) formed with embedded electronic components has attracted considerable interest because of its simple and inexpensive micro-fabrication technology, along with the following additional benefits: (a) low production cost; (b) optical transparency; (c) biocompatibility; (d) excellent contour accuracy; and (e) easy bonding to other substrates [30,31,32]. On the other hand, the elastic properties of PDMS are strongly dependent on the amount of cross-linking agent, UV exposure time and wall thickness [32,33]. As a result, the deformation properties of PDMS microfluidic devices fabricated from soft PDMS change their flow behaviors, which can sometimes lead to ambiguous results [33,34,35]. Although embedded obstacles in PDMS microfluidic channels are used widely in many experimental applications, validation of the deformable microfluidic channels to the flow behavior in steady-state flow has not been investigated. Therefore, this limited verification gives rise to questions as to whether the experimental conditions were reliable, and if the influences of the flow rate, microfluidic channel geometry and material property are significant factors for the flow behavior.

In the present article, the flow was discharged completely from the outlet and took several minutes to reach steady-state flow under low flow rates. This study had two research purposes. The first was to introduce a change in the cross-sectional area during steady-state flow and compare it with quasi-steady flow under low flow rates. Many researchers have utilized various PDMS material properties and focused on high flow rates [36,37], but very few studies have dealt with low flow rates. In fact, microfluidic devices can be operated at low flow rates due to their specific uses, and this comparison suggests that such an intensive study of this topic might be beneficial to researchers. The experimentally-observed PDMS bulging was compared with the numerically predicted values. Numerical analyses verified by experimental results allow researchers to provide more practical information with respect to the optimal design and operating conditions of microfluidic devices because many applications with microfluidic devices are operated with steady-state flow. The second goal was to investigate the changes of flow velocity and pressure drop using a range of experimental conditions because many chemical reactions in microfluidic channels require accurate control. To the best of the authors’ knowledge, this is the first study to compare the difference between quasi-steady-state flow and steady-state flow with different characteristic features of a short length of PDMS microfluidic channels.

## 2. Results and Discussion

**Figure 1:**
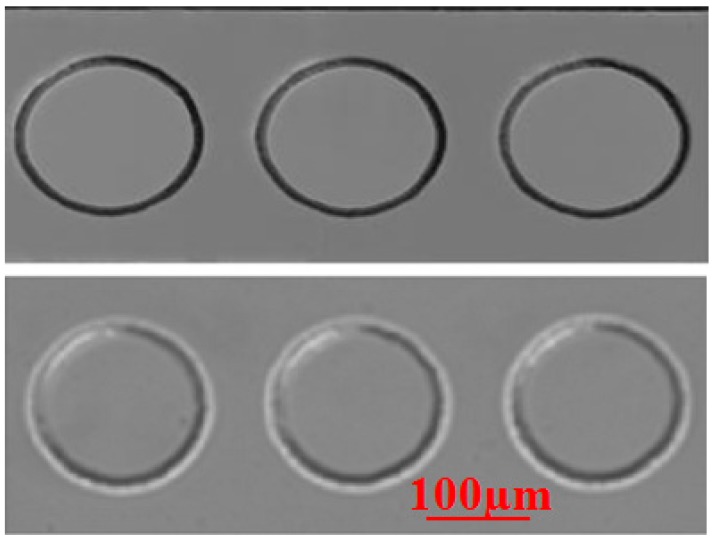
Close-up image of the obstacles with no flow and high flow rate (4.0 µL/min).

Figure 1 shows the expansion of shallow PDMS microfluidic channels (15 µm) with applied flow rates using a 100 µM solution of Rhodamine 6G dye in comparison with the no flow rate (top) and 4 µL/min (bottom) when liquid flows through microfluidic channels. In the previous study, a significant bulging was observed when the flow initially contacted the microstructure [24]. The experimental observation proved that serious discrepancies from the predicted values arose during quasi steady-state flow condition. Significantly less bulging compared to quasi steady-state flow was observed when the flow reached the steady-state condition. The steady-state flow operation suffers much less from the effect from PDMS bulging and provides a more reliable experimental result. However, it did not disappear during microfluidic device operation. This indicated that PDMS bulging, which causes changes in the cross-sectional areas, affects the flow behavior. Actually, many applications of microfluidic devices can be operated at low flow rates due to their specific uses, and focused on steady-state flow conditions but most cases neglect PDMS bulging. Therefore, proper selection of flow condition (*i.e.*, steady-state flow) for a microfluidic device is required for future applications because the PDMS bulging phenomenon has a strong interconnection to flow velocity and pressure drop.

**Figure 2:**
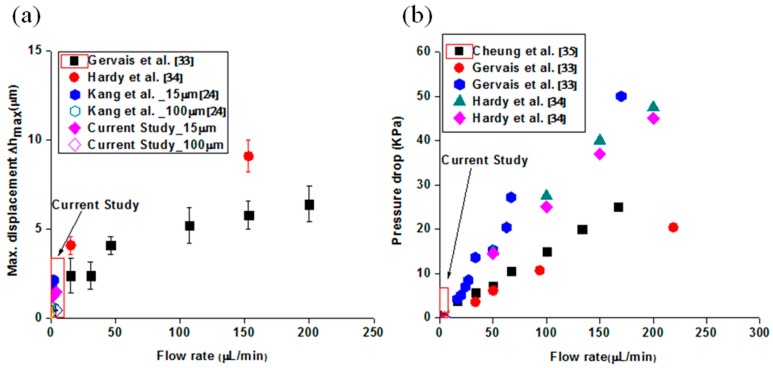
(**a**) Analysis of PDMS bulging with several references (Red box: current study); (**b**) Comparison of pressure drop analysis in the PDMS microfluidic channels.

Figure 2a presents a comparison of PDMS bulging with several studies. It was manifest that PDMS bulging was a function of the applied flow rates. It means that increasing flow rates causes large PDMS bulging, as shown in Figure 2a and directly affect the pressure drop. Generally, the influence of PDMS bulging on the pressure drop has attracted considerable interest and been observed by several research groups, as shown in Figure 2b. The relationship between the flow rate and pressure drop is linear based on classical theory of fluid mechanics, but those reference studies showed a non-linear relationship due to PDMS bulging. This study could be a meaningful study because it deals with the flow velocity and pressure drop when the microfluidic channels are operated at low flow rates. However, a limited number of information sources make it difficult to derive meaning information.

**Figure 3:**
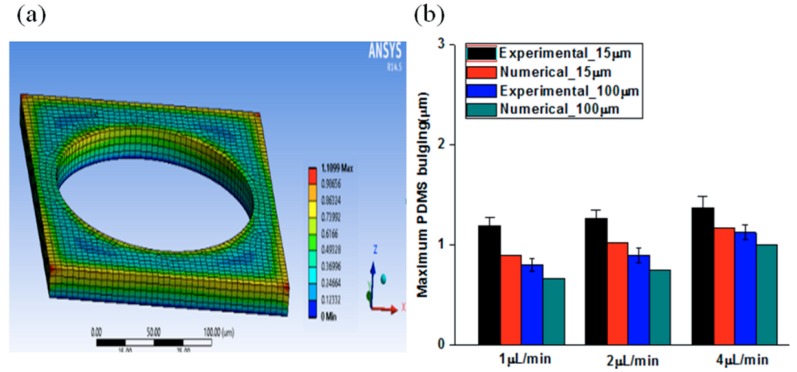
(**a**) Geometry and mesh in ANSYS Workbench for PDMS bulging (15 μm channel depth); (**b**) Comparison of numerical and experimental PDMS bulging (mixing ratio = 10:1).

The numerical analysis using ANSYS Workbench was used and illustrated the geometry and mesh, as shown in Figure 3a which used a simple 3D modeling. The same principle was also applied to 100 µm microfluidic channel depth for the investigation of the influence of microfluidic channel depth. The results showed the distribution of PDMS bulging in which the position was far away from the bottom surface exhibited relatively large bulging. A comparison of PDMS bulging performed by numerical and experimental results is shown in Figure 3b. The discrepancy became a significant problem when increasing flow rates and shallow microfluidic channels induced unexpected PDMS bulging. It means that flow rates and microfluidic channel geometry are important factors to achieve meaningful information. The reason for this discrepancy was that the Young’s modulus and Poisson ratio used in this simulation did not represent the actual PDMS properties. In spite of these drawbacks, the ANSYS Workbench analysis in this study showed good agreement with the experimental analysis (mixing ratio = 10:1) and increased reliability through actuated mesh controls.

**Figure 4:**
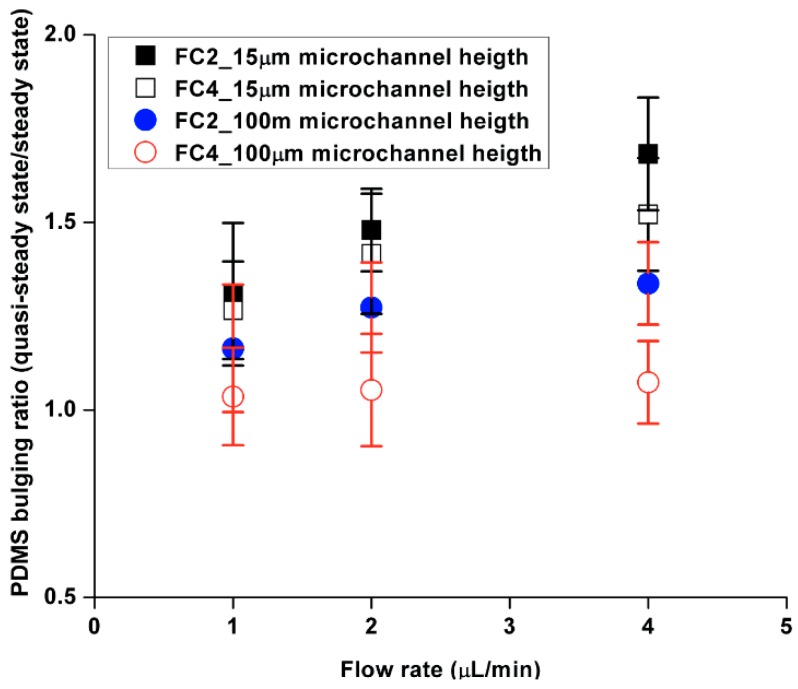
Comparison of the PDMS bulging ratio as a function of flow rates (PDMS bulging of quasi-steady state flow to steady-state flow).

Figure 4 presents the results of an analysis of the maximum displacement between quasi-steady-state flow during transient filling and steady-state flow after filling flow originating from the outlet. A change in large cross-sectional areas was observed when the flow initially contacted the embedded obstacles under quasi-steady-state flow conditions. On the other hand, when the flow became steady-state, the change in cross-sectional areas decreased and maintained constant values. An analysis of the PDMS bulging ratio which was defined as bulging of quasi-steady flow to steady-state flow, as shown in Figure 4, was between 25%~65% in the case of the 15 µm microfluidic channels. However, the values of difference were approximately 5%~35% in the case of 100 µm microfluidic channels. This suggests that: (1) a shallow microfluidic channel leads to a relatively large discrepancy from steady-state flow, and (2) increasing flow rates increase the discrepancy between quasi-steady state and steady state flow. This demonstrates that an appropriate microfluidic channel geometry and applied flow rate are important factors to determine trustful results. However, the influence of selection on steady-state flow during the experiment has not been considered an important factor, but it is one of the control factors that generate reliable data.

**Figure 5:**
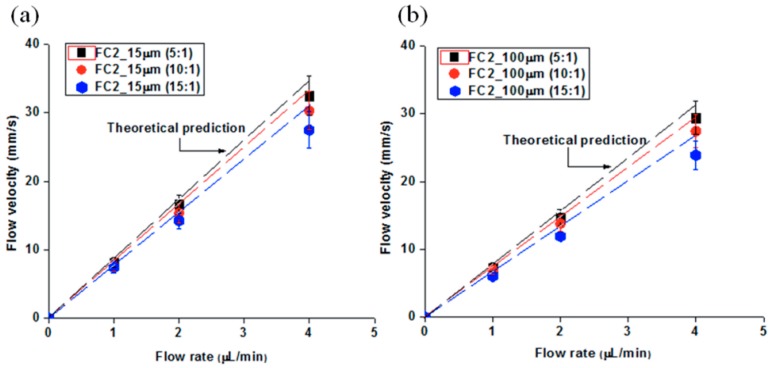
(**a**) Flow velocity analysis as a function of the flow rates in 15 µm microfluidic channels; and (**b**) Flow velocity analysis as a function of the flow rates in 100 µm microfluidic channels.

Figure 5 presents a comparison of the experimental and theoretical flow velocity in microfluidic channels using theoretical Equations (3)–(6), which were formulated using different mixing ratios of PDMS solutions. Owing to the polymerization of the PDMS, soft material properties were observed in the case of a high portion of PDMS monomer and a low portion of curing agent in the PDMS mixture. The soft microfluidic channels using a mixing ratio of 15:1 induced larger PDMS bulging compared to microfluidic channels which had a small amount of cross-linking agent in the PDMS mixture. As expected, a large discrepancy of the flow velocity between the theoretical and experimental results was observed in the soft PDMS microfluidic channels, indicating that the flow velocity was dependent on the material properties. The largest discrepancy observed in the case of FC2 (channel drpth: 15 µm, mixing ratio: 15:1) at 4.0 µL/ was about 17%. The discrepancy in this study became an issue when the microfluidic devices were operated under the following conditions: (1) highest flow rate (*i.e.*, 4 µL/min); (2) shallow microchannel (*i.e.*, 15 µm); and (3) soft PDMS (*i.e.*, mixing ratio = 15:1). An analysis of flow velocity, as shown in Figure 5b indicated a relatively good agreement with the classical theory compared to Figure 5a. Increasing the microfluidic channel depth induces less PDMS bulging, which causes a small change of flow velocity. The result in Figure 5b, however, shows that PDMS bulging should not be ignored, and that it can become a significant factor when high applied flow rates operate in soft, shallow microfluidic channels and less curing agent is used. The increment of cross-sectional areas due to PDMS bulging results in a decrease in flow velocity from the predicted values. The increasing discrepancy from the predicted value led to a reduced reliability of the microfluidic device because the friction factor and Reynolds number are a function of the flow velocity.

**Figure 6:**
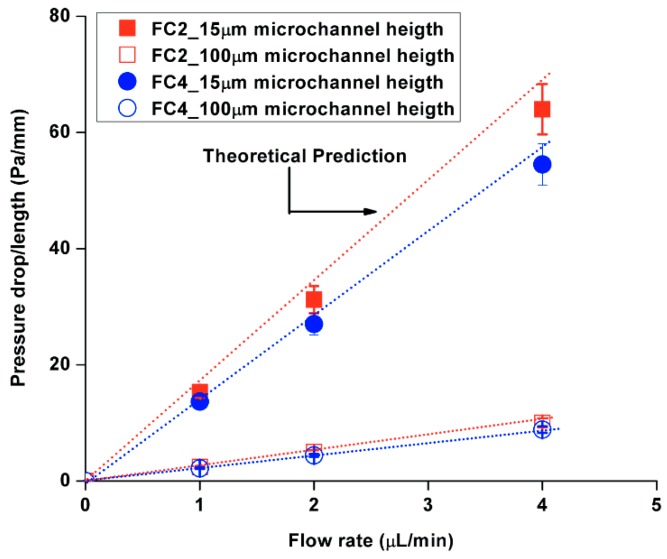
Comparison of the pressure drop/length analysis in 15 µm and 100 µm microfluidic channels.

Figure 6 shows the results of an investigation of pressure drop using two different diameters of embedded obstacles and microfluidic channel height. The experimentally measured pressure drop data of fluid-flow through a porous medium was compared with the theoretical results using Equations (7)–(10). The Δp/L between the theoretical and experimental results showed relatively good agreement, whereas the discrepancy began to increase under the following conditions: (1) increasing flow rates; and (2) embedded obstacles with a larger diameter. The importance of flow rates was already observed in the previous result, but the embedded obstacles also contributed to the discrepancies from the predicted values as the diameter size increased. Regarding the flow velocity, the pressure drop investigations showed that the experimental results were lower than the predicted values due to PDMS bulging.

## 3. Materials and Methods

### 3.1. Procedures

**Figure 7:**
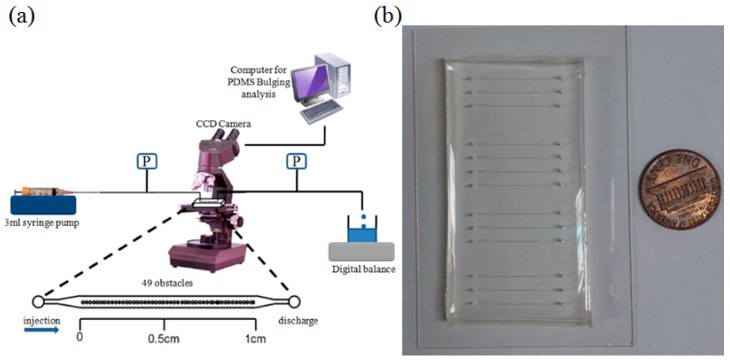
(**a**) Schematic diagram of the experimental apparatus; (**b**) Actual image of the microfluidic devices.

A liquid food dye solution (ESCO Foods Inc., San Francisco, CA, USA) was utilized for these experimental investigations of the flow properties. The density and viscosity was characterized at room temperature by a tensiometer (KSV Sigma 702, Scientific Solutions, North Chelmsford, MA, USA) and viscometer (NametreViscoliner, Lowell, MA, USA). The measured density was 0.685 ± 0.006 g/cm^3^, while the viscosity was determined to be 1.5625 ± 0.0016 cp. The variation of temperature along the microfluidic channels was less than 0.1 °C, which matched well with other studies where the flow throughout microfluidic channels was constant because the measured temperature change inside microfluidic devices showed less than 0.1 °C difference, which agreed with the reference study [38]. Therefore, the properties of the fluid were assumed to be constant at the room-temperature which did not influence on the change of viscosity and density. Figure 7a illustrates a schematic diagram of the experimental setup with circular obstacles in the microfluidic channels. 

AutoCAD software (AutoDesk Inc., San Rafael, CA, USA) was used to produce a mask design which was then printed on a transparent film by CAD/Art Service Inc. (Bandon, OR, USA). The silicon wafer fabrication process by the normal wet etching process. A positive photoresist (AZ P4620) was spin-coated onto a 4 inch silicon wafer (Silicon Quest, Santa Clara, CA, USA) for shallow microfluidic channels (*i.e.*, height: 15 μm), whereas a negative photoresist was used for thick microfluidic channels (*i.e.*, height: 100 μm). Photoresist development using appropriate UV exposure allowed the microfluidic channel design to be transferred to silicon wafers which produced molds of the correct desired height. Each microfluidic chip was fabricated with three different mixing ratios of PDMS (PDMS monomer: curing agent = 5:1, 10:1, 15:1) which was then poured onto the wafer inside the mold structure to make a 5 mm thick chip with microstructure to be characterized. The microfluidic channel was cured at 85 °C for 1 h to increase PDMS cross-linking and peeled off the wafer to make inlet and outlet ports using a 19 gauge punch (Technical Innovation, Brazoria, TX, USA). The microfluidic chips contained fabricated channels of a measured width equal to 243 ± 1 μm and a measured height of 15 ± 1 and 100 ± 5 μm. The PDMS and glass slide (Fisher Scientific, Pittsburgh, PA, USA) was exposed to oxygen plasma (Plasma Cleaner PDC-326, Harrick Plasma, Ithaca, NY, USA) to make a hydrophilic surface. An aligned row of periodic obstacles was arranged along the centerline of each microfluidic channel. The micro-fabricated obstacles in this study had diameters of 172 μm (*i.e.*, FC2) and 132μm (*i.e.*, FC4). Tygon tubing connected a syringe pump to the microfluidic chip at the microfluidic chip inlet. The flow rates were maintained constant using a syringe pump (HA1100WD, Harvard Instrument, Holliston, MA, USA) operating at 1.0, 2.0 and 4.0 μL/min. The pressure variation was pre-tested and the accuracy was ±0.5% of the original pressure. The flow velocity and pressure drop were captured after the flow achieved a steady-state along with PDMS bulging. The PDMS microfluidic channels were measured by confocal microscopy (A1+, Nikon Instech Co., Ltd., Tokyo, Japan; Figure 1b) recorded using an image-intensifying CCD camera (Digital Sight DS-Q1MC, Nikon). The PDMS bulging was mainly focused on steady-state flow but the liquid food dye solution that initially contacted the embedded obstacles was also observed to show the characteristics of the fluid flow. The 3-D image files recorded on a computer (see Figure 1a) enabled a direct determination of maximum PDMS bulging using the Imaris 7.2.3 image-processing software (Bitplane Inc., Concord, CT, USA). The pressure drop data was measured directly in each experiment using a gauge pressure transducer (PX138, Omega Engineering Inc., Stamford, CT, USA). The transducer voltage was digitized and recorded with a computerized data acquisition system (DI-148U, DATAQ Instrument, Akron, OH, USA). The flow velocity was measured using a digital balance when the fluid passing through the microfluidic channels was collected in the test section to be weighed; it takes time to reach steady-state flow. The data in this study was captured after steady-state flow had been achieved. This experiment does not deal with the effects of surface roughness because the average roughness featured in this study was small (<20 nm) and was operated at very low Reynolds numbers in the microfluidic channels.

### 3.2. Data Analysis

The measured pressure, P_System_, in the microfluidic system during the process of filling an initially empty microchannel is composed of pressure drops through the chip connection tubing (P_Tubing_), chip injection needle (P_Needle_), chip entrance region (P_Entrance_), obstacle region along the microchannel (P_Obstacles_), capillarity effects due to the fluid-free surface (P_Cap_), and the microchannel exit losses (P_Exit_) [24]. Therefore:

P_System_ = ∆P_Tubing_ + P_Needle_ + P_Entrance_ + ∆P_Obstacles_ + ∆P_Cap_ + ∆P_Exit_ (1)

When the fluid front has not reached the end of the microchannel, the pressure drop due to the air exiting the microchannel in front of the liquid flow is negligible at the low flow rates used in this study. The total pressure needed to drive the fluid through the apparatus tubing, injection needle, microfluidic channel entrance region, and to overcome microfluidic channel capillarity effects up to when the fluid just reaches the first obstacle can be measured experimentally using a pressure transducer when the fluid initially contacts the first obstacle. The pressure drop associated with the capillarity effects, P_Cap_, at the liquid-free surface is a large and non-negligible component of P_System_. Because fully developed steady-state flow was the main focus in this study, the right hand pressure except for P_Obstacles_ must be subtracted from the measured pressure. The capillary pressure drop inside the rectangular microchannels can be calculated using the following equation [39]:

(2) $$\Delta p=\frac{2(H+W)\sigma \cos\Theta }{HW}$$

where *H* is the channel height, *W* is the channel width, σ is the liquid-air surface tension, and Θ is the liquid contact angle with the surfaces. The contact angles measured during filling of the microfluidic channels in this study varied from 58°–79° which was dependent on channel depths. It may be that the diffusion of oxygen molecules by plasma did not effectively penetrate on shallow microchannels. The capillary pressure drops in this study were 1.61~2.23 kPa.

To verify the experimental deviations from the steady-state flow theories, the actual flow velocity was determined by measuring the weight of water in the collecting container, represented by Equations (3)–(5), whereas theoretical flow velocity was determined by Equation (6) [27]. The difference between Equations (5) and (6) is caused by the changes in cross-sectional areas:

(3) $$v_{Experimental\;mean}=\frac{Q_{disch\arg e}}{A}=\frac{Q_{disch\arg e}h_{actual}}{V_{actual}}$$

(4) $$Q_{disch\arg e}=\frac{V_{fluid}}{t}=\frac{m_{fluid}}{\rho \times t}=\frac{\dot{m}}{\rho },\;\dot{m}=\frac{m}{\Delta t}$$

(5) $$v_{Experimental\;mean}=\frac{\dot{m}h_{actual}}{V_{actual}\rho }$$

(6) $$v_{Theoretical\;mean}=\frac{Q}{A_{Theoretical}}$$

where $\dot{m}$ is the mass of water measured over the time period, *t* is time, ρ is the density of the flow, *h* is the height of the channel, and *A* is the cross-sectional area. The pressure drops of fully developed, steady-state and incompressible flows through microfluidic channels can be calculated using Darcy-Weishbach relation [40,41]:

(7) $$\frac{dp}{dz}\approx \frac{\Delta P}{L}=f\frac{\rho \bar{V^{2}}}{2\varepsilon^{2}{\bar{D}}_{h}}$$

where *L* is the channel length, $\bar{D_{h}}$ is the mean hydraulic diameter, ε is the porosity of the medium, *f* is the Darcy friction factor, and $\bar{V}$ is the volume-averaged velocity, respectively. The mean hydraulic diameter can be defined as [42]:

(8) $$\bar{D_{h}}=4\frac{Cross\;\sec tional\;area}{wetted\;perimeter}\times \frac{L}{L}=4\frac{Volume\;available\;flow}{Total\;wetted\;surface}$$

The detail geometry of microfluidic channels was listed in Table 1. The Darcy friction factor for rectangular microchannels is a function of the channels’ aspect ratio (α = w/h, where w: width, h: height) of the rectangular microchannels. Α varies from 56.9 for square channels to 96 for narrow rectangular microchannels [42]. In addition, the Darcy friction factor results from the present study show that the relative trend values agree well with the theoretically expected values at the very low Reynolds numbers investigated here:

(9) f=96Re

(10) Re=Dh¯ρV¯μ

### 3.3. Finite Element Model

The interaction between liquid flow and PDMS microchannel were performed using commercial software (ANSYS Workbench, Canonsburg, PA, USA) to compare the experimental measurement. An AutoCAD file was used to describe the general geometry in this study. The microchannel structures for the analysis of steady-state flow deformation had two different heights (15 µm and 100 µm) and a width of 243 µm. The refinement of 3D mesh increased the relevance for the finite element method which changed the default mesh to decrease mesh sizing. The modification of mesh size provided more reliable data in ANSYS Workbench [43]. The classical material properties of PDMS were used in this simulation because this study did not perform any measurements of PDMS properties. The value of the Poisson ratio was 0.49 and the Young’s modulus was assumed to be 750 kPa [44]. It is known that PDMS properties such as Poisson ratio and Young’s modulus are dependent on cross-linking agent, UV exposure time, and curing temperature. The boundary condition in this study was that both sidewalls were set as under free boundary conditions and the bottom wall which was strongly bonded with glass slide was set as the fixed boundary condition. The measured pressure was then applied to the PDMS structure in the ANSYS simulation and compared with the experimentally determined deformation. A mesh refinement indicated that a mesh of 23,947 nodes and 4,620 elements would produce a reliable result in the current structure.

## 4. Conclusions

The characteristic features of PDMS bulging in microfluidic channels are a function of the applied flow rate, operation flow conditions, microfluidic channel geometry (*i.e.*, channel height and diameter of obstacles) and mixing ratios of PDMS mixture when the fluid-flow was discharged completely from a porous medium. Although this experiment was operated with fully developed, steady-state and incompressible flows, the cross-sectional change due to PDMS bulging yielded distorted flow behaviors, such as changes in pressure drop and flow velocity, from the predicted values. Steady-state flow induced considerably less PDMS bulging compared to quasi-steady flow. Three-dimensional finite element modeling using ANSYS workbench supported the characteristic features of PDMS bulging under steady-state flow condition. Therefore, the flow behaviors in steady-state flows can follow the classical theory more easily than can quasi-steady flow. The comparison of experimental results with the theoretical data showed that the measured flow velocity and pressure drop were lower than the predicted values and the discrepancy was a function of the following four conditions:

(1) The geometry of the microfluidic channels: 15 µm microfluidic channels generated an unexpected discrepancy in the flow velocity between the theoretical and experimental results compared to the 100 µm microfluidic channels. In addition, the diameter of the embedded obstacles (*i.e.*, FC2 and FC4) influenced the flow behaviors. For example, large embedded obstacles (*i.e.*, FC2) caused a substantial discrepancy from the predicted values.
(2) Operational flow conditions: The flow operated in steady-state induced much less PDMS bulging and followed the classical theory compared to quasi-steady state flow.
(3) Applied flow rates: sizeable PDMS bulging was observed at high flow rates, and the discrepancy from the predicted values became severe as the flow rates increased.
(4) PDMS mixing ratios: The PDMS mixing ratios were found to be a controlling factor in changing the material property. Increasing stiffness by increasing curing agent (B) led to the following of the theoretical values of the flow velocity and pressure drop, whereas PDMS microfluidic channel by decreasing curing agent began to generate a noticeable discrepancy.

As a result, proper selection of operation conditions, flow rates, geometry of the microfluidic channel, and the material properties play key roles in obtaining more reliable experimental results.

## Figures and Tables

**Table 1:** Geometry of microfluidic channels.

Obstacle and Depth	Diameter of Obstacle (mm)	Overall Microchannel Length (mm)	Mean Porosity (%)	Mean Hydraulic Diameter (µm)
FC2_15 µm	0.172	10.1	54	23.7
FC4_15 µm	0.132	9.14	70	25.2
FC2_100 µm	0.172	10.1	54	71.9
FC4_100 µm	0.132	9.14	70	88.5
